# Supplementary material for: OCT4 impedes cell fate redirection by the melanocyte lineage master regulator MITF in mouse ESCs
Source: Nat Commun. 2017 Oct 18;8:1022. doi: 10.1038/s41467-017-01122-1 (PMC5647326; doi:10.1038/s41467-017-01122-1)
Supplement: Supplementary file 1 — Supplementary Information [file 41467_2017_1122_MOESM1_ESM.pdf]

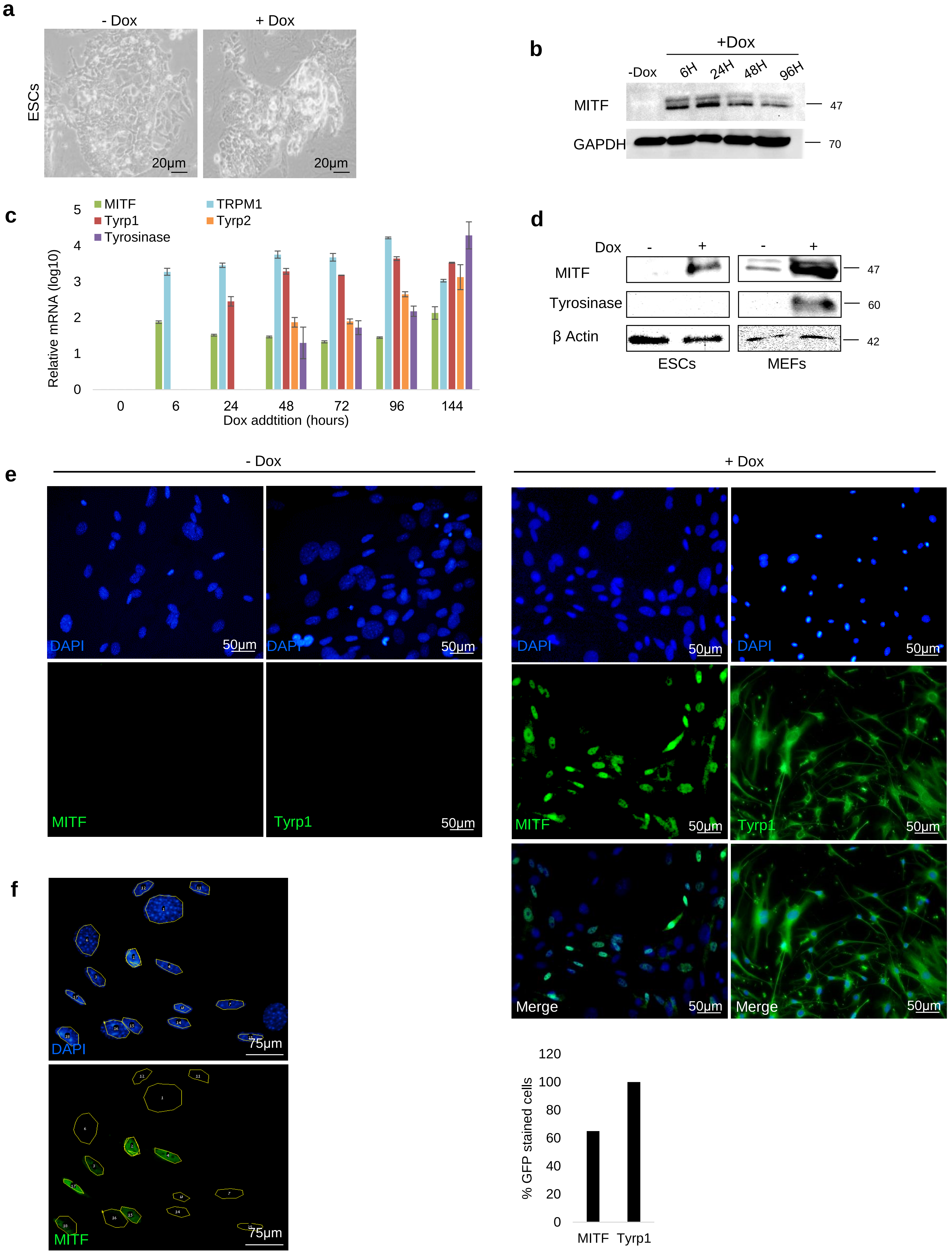

Supplementary Figure 1 : MITF efficiently transdifferentiates MEFs.

Supplementary Figure 1: MITF efficiently transdifferentiates MEFs. (a) Brightfield images of ESCs prior to Dox induction and at day 12 post Dox induction. This was one of n=3 experiments (b) MITF protein levels in MEFs at different time points post Dox induction. (c) *Mitf*, *Tyrp1*, *Tyrosinase*, *Trpm1*, and *Tyrp2* mRNA levels in MEFs at different time points post Dox induction compared to vehicle-treated cells. Levels were normalized to *Gapdh* and fold changes relative to control are shown. Error bars represent ± SEM. * indicates $p < 0.05$ ($n = 3$). (d) MITF and TYR protein levels in *Mitf* knock in ESCs and *Mitf* knock in MEFs at day 12 post Dox induction (e) Immunostaining of MITF (green) and TYRP1 (green) in MEFs at day 12 post Dox induction. DAPI-stained nuclei appear blue. This was one of n=3 experiments (f) Green pixel quantification for MITF-positive cells compared to DAPI-stained nuclei was made using ImageJ software and quantification for TYRP1-positive staining was made by counting the whole field. An average number of 30 cells were taken from each field, with two fields analyzed for each group.

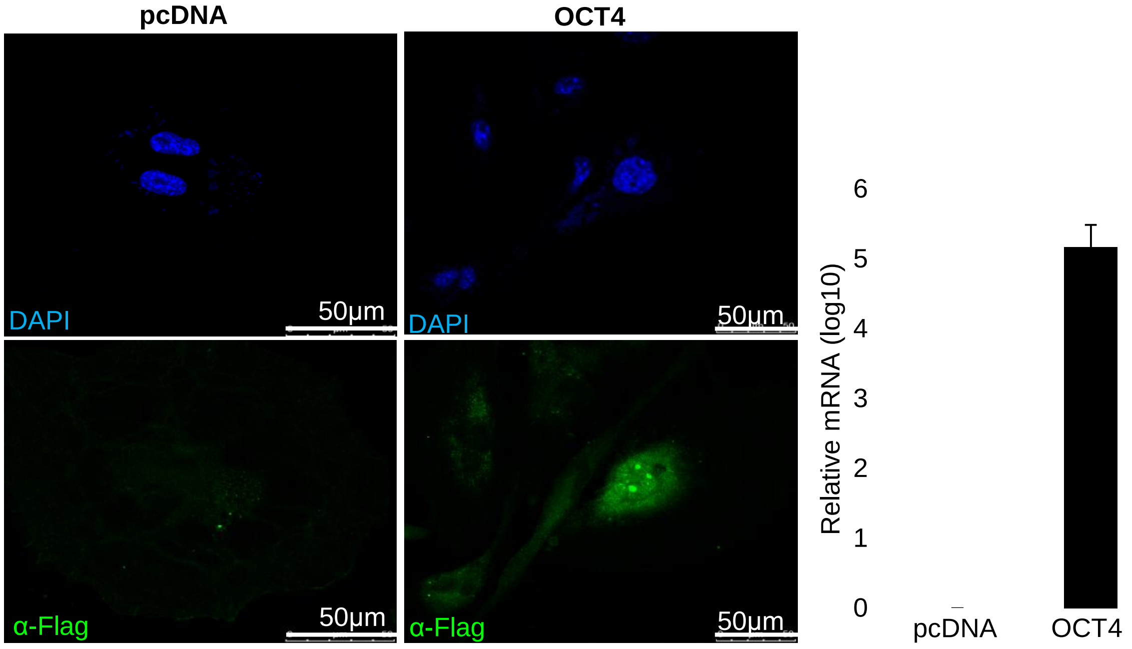

Supplementary Figure 2: OCT4 impedes mESCs differentiation despite MITF expression. MEFs were transfected with a plasmid for expression of OCT4 or empty vector as control. Left: Immunostaining of OCT4-flag (green) performed at day 6 post Dox induction. DAPI-stained nuclei appear blue (left panel). This was one of n=2 experiments. Right: *OCT4* mRNA level at day 6 post transfection. Levels were normalized to *Gapdh*, and fold changes relative to control are shown. Error bars represent ± SEM. * indicates $p < 0.05$ ($n = 3$). Error bars represent ± SEM.

Supplementary Figure 2 : OCT4 impedes mESCs differentiation despite MITF expression.

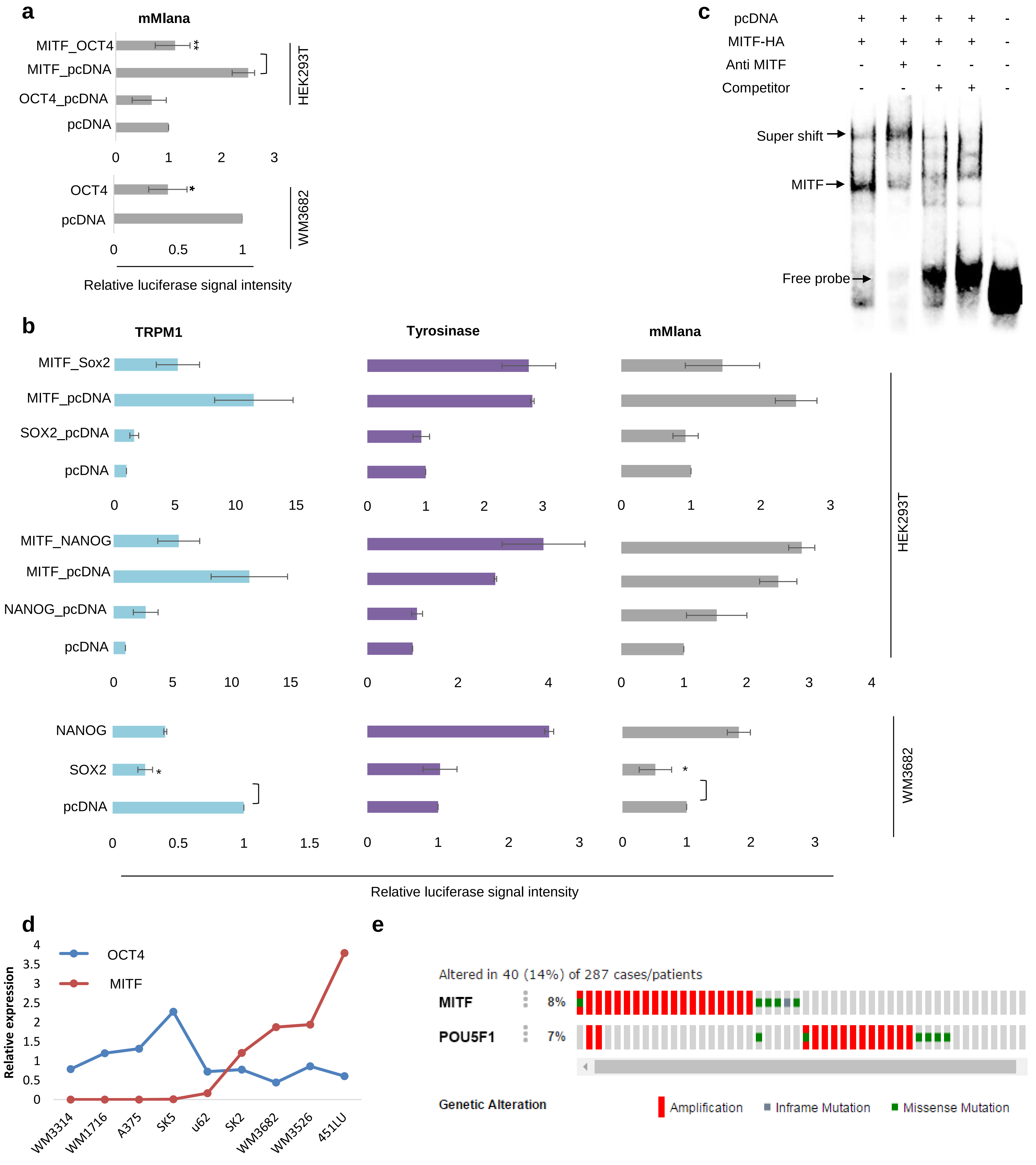

Supplementary Figure 3: OCT4 interferes with MITF transcriptional activity

Supplementary Figure 3: OCT4 interferes with MITF transcriptional activity. (a) Upper: HEK293T cells were co-transfected with luciferase reporter driven by the m*MLANA* promoter and with plasmids for expression of MITF, OCT4, or empty plasmid as control. Lower: WM3682 cells were co-transfected with luciferase reporter driven by the m*MLANA* promoter and with either OCT4 expression plasmid or empty plasmid as control. Luciferase activity was normalized to *Renilla* luciferase activity. Fold changes relative to control are shown. Error bars represent ± SEM, * indicates $p < 0.05$ ($n = 3$). (b) Upper: HEK293T cells were co-transfected with luciferase reporter driven by *TRPM1*, *TYR*, or *mMLANA* promoter and with plasmids for expression of MITF, SOX2, or NANOG or empty plasmid as control. Lower: WM3682 cells were co-transfected with luciferase reporter driven by *TRPM1*, *TYR*, or m*MLANA* promoter and with SOX2 or NANOG expression plasmids or empty plasmid as control. Luciferase activity was normalized to *Renilla* luciferase activity. Fold changes relative to control are shown. Error bars represent ± SEM, * indicates $p < 0.05$ ($n = 3$). (c) A probe corresponding to the E-box region of the *TRPM1* promoter was used in EMSA to test *in vitro* binding of MITF to this sequence. Polyclonal anti-MITF antibody was used for supershift analysis. MITF bound probes and free probe are marked with arrows. This was one of n=3 experiments. (d) *MITF* and *OCT4* expression in melanoma cell lines. This was one of n=2 experiments (e) Visualization of genetic alterations in melanoma related to MITF and OCT4 based on TCGA database using cBioPortal site for Cancer Genomics: http://www.cbioportal.org/.

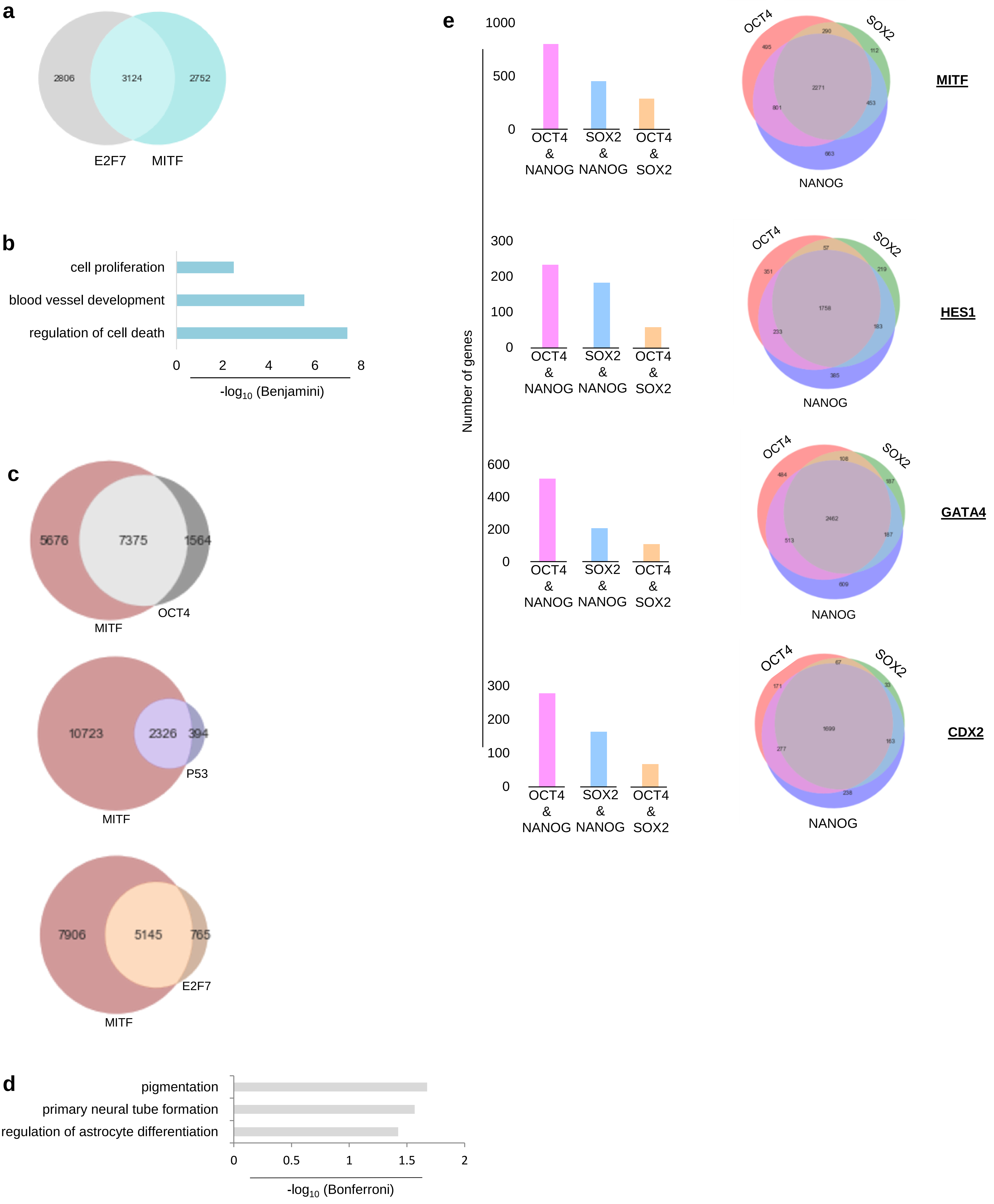

Supplementary Figure 4: Overlapped ChIP-seq peaks

Supplementary Figure 4: Overlapped ChIP-seq peaks. (a) Venn diagrams representing the number of unique gene promoters bound by E2F7 and MITF (ChIP-seq originated from melanoma cells) and the overlap between them based on ChIP-seq peaks. (b) A view of the selected biological pathways enriched within the overlapped gene set. (c) Venn diagrams representing the number of unique gene promoters and the overlap of MITF (ChIP-seq originated from normal melanocytes) with OCT4, P53, and E2F7. (d) Selected biological pathways identified by GO enrichment analysis of genes bound by both OCT4 and MITF. (e) Right: Venn diagrams representing the number of gene promoters bound by OCT4, SOX2, NANOG, and the specified lineage transcription factors and the overlap between them based on ChIP-seq peaks. Left: Number of gene promoters that overlap between the combinations for each pair of OCT4, SOX2, and NANOG.

a

Figure 1b:

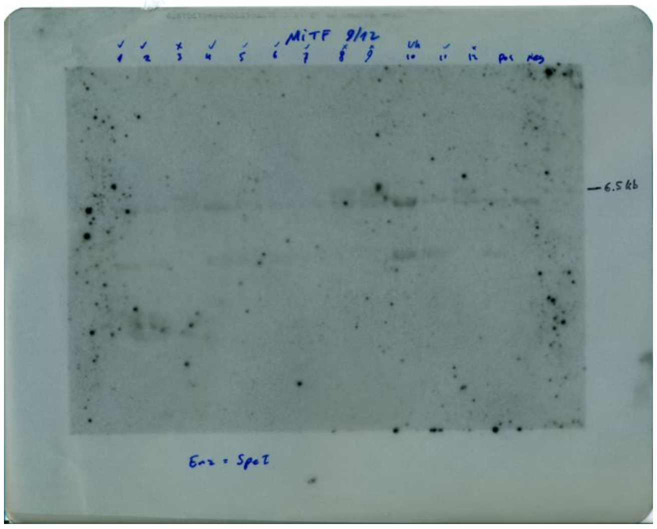

b

Supplementary Figure 1b:

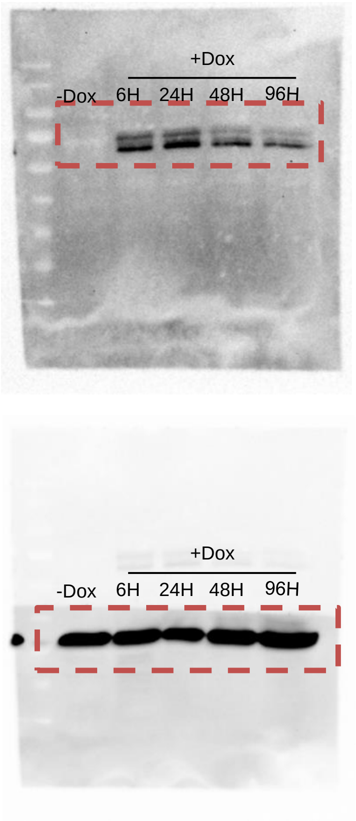

c

Supplementary Figure 1d:

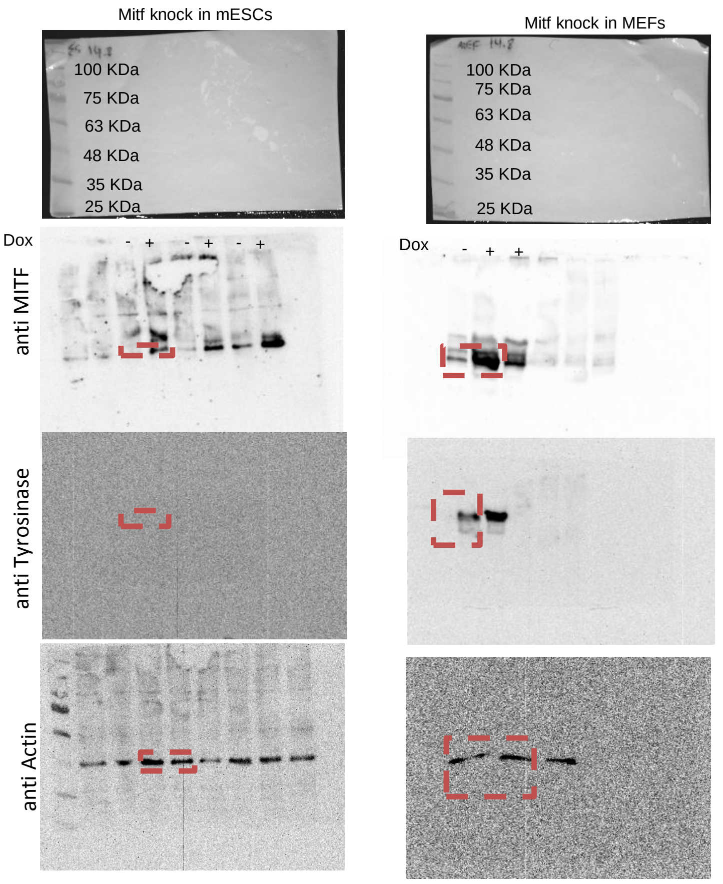

d

Figure 2b:

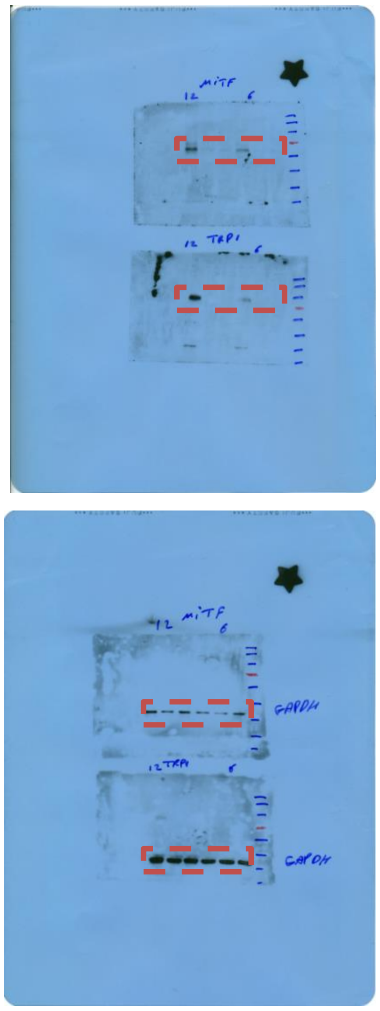

Supplementary Figure 5

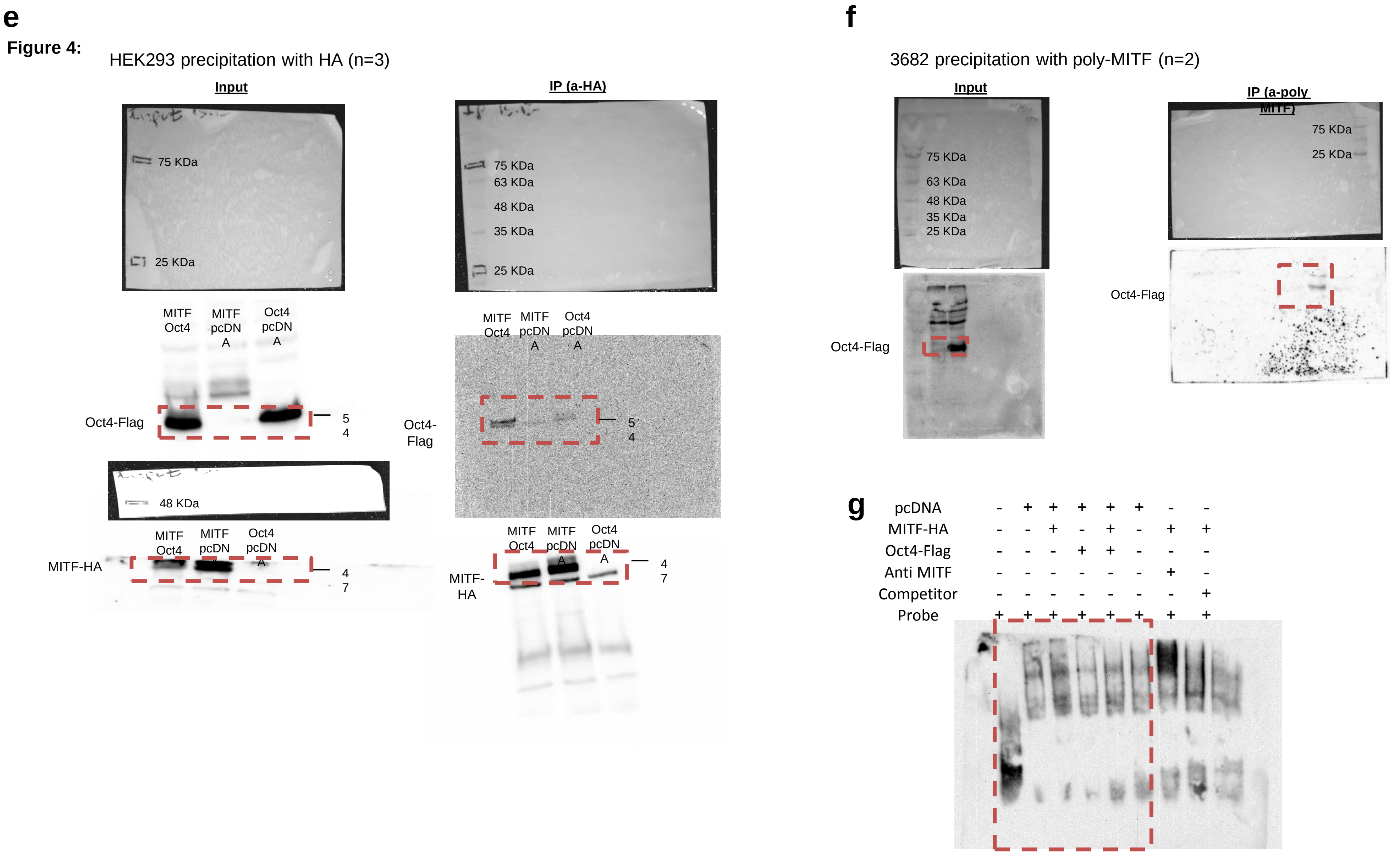

Supplementary Figure 5: Uncropped scans of blots.

(a) Uncropped Southern blot found in Figure 1b. Southern blot verification of tetO-Mitf knock in to mESCs. Line 10 represent the clone that was correctly targeted and was used for injection into E3.5 blastocyst to obtain ROSA26-M2RtTa+/+mCol1a-TetO-Mitf+/- derived MEFs and somatic cells. This was one of n=3 experiments.

(b) Uncropped Western blot found in Supplementary Figure 1b. MITF protein levels in MEFs at different time points post Dox induction. This was one of n=1 experiments.

(c) Uncropped Western blot found in Supplementary Figure 1d. MITF and TYR protein levels in Mitf knock in mESCs and Mitf knock in MEFs at day 12 post Dox induction. This was one of n=3 experiments.

(d) Uncropped Western blot found in Figure 2b. MITF and TYRP1 protein levels in Mitf knock-in MEFs at days 6 and 12 post Dox induction. This was one of n=3 experiments.

(e) Uncropped Western blot found in Figure 4f. Co-IP assay of MITF-HA and OCT4-flag in HEK293T cells. This was one of n=3 experiments.

(f) Uncropped Western blot found in Figure 4g. Co-IP assay of endogenous MITF and OCT4-flag in WM3682 melanoma cells. This was one of n=2 experiments.

(g) Uncropped Western blot found in Figure 4h. EMSA assay which was conducted using a probe corresponding to the E-box region. This was one of n=3 experiments.

Supplementary Table 1. Sperman Correlation

Sperman Correlation Pval

d_1	d_2	d_3	d_4	d_5
	Primary Melanocyte	MEF +DOX	MEF - DOX	MEF
MEF +DOX	0	1	0	0
MEF - DOX	1.26686E-24	0	1	0
MEF	0	0	0	1
Primary Melanocyte	1	0	1.27E-24	0

Sperman Correlation coef

d_1	d_2	d_3	d_4	d_5
	Primary Melanocyte	MEF +DOX	MEF - DOX	MEF
MEF +DOX	0.261387367	1	0.792906	1
MEF - DOX	0.048178307	0.792906	1	1
MEF	0.215945725-	0.553468	0.729358	1
Primary Melanocyte	1	0.261387	0.048178	0-

Supplementary Table 2. Oligonucleotides Used in This Study, Related to Experimental Procedures

Real-Time PCR Primers		nucleotide sequence
Primers list used for qRT-PCR (written from 5' to 3'):		
Mouse:		
Oct4 (pou5f1)	Forward	GCTCACCCTGGGCGTTCTC
	Reverse	GGCCGCAGCTTACACATGTTC
Sox2	Forward	ACAGATGCAACCGATGCACC
	Reverse	TGGAGTTGTACTGCAGGGCG
Nanog	Forward	CCTCCAGCAGATGCAAGAACTC
	Reverse	CTTCAACCACTGGTTTTTCTGCC
MITF	Forward	GAAACCTTGCTATGCTGGAAATG
	Reverse	GAGCTTGCTGTATGTGGTACTT
Tyrosinase	Forward	ACCCAGTATGAATCTGGATCAATG
	Reverse	CTGTGAGTGGACTGGCAAAT
Tyrp1	Forward	GGTCTTTGACGAATGGCTAAGG
	Reverse	CCAGAATGGCACCATGTTGTA
Tyrp2	Forward	CTTCAACCGGACATGCAAATG
	Reverse	CCGGCTTCTTCCGATTACAG
TRPM1	Forward	CTCCCGAAGCTCTTGATATCTG
	Reverse	AGCCTTGATCAGACCTTTCC
GFAP	Forward	GGTTGAATCGCTGGAGGAG
	Reverse	TGCTCCCGGAGTTCTCG
Troponin	Forward	GACTTTGATGACATCCACAGGA
	Reverse	AGCTCCTCTTCCTCCTTCTT
Desmin	Forward	CATCTCTGAGGCTGAAGAATGG
	Reverse	CAGCGCATCGTTGTTCTTATTG
Nestin	Forward	TTGCAGACACCTGGAAGAAG
	Reverse	CCTCTGGTATCCCAAGGAAATG
GAPDH	Forward	TTCACCACCATGGAGAAGGC
	Reverse	CCCTTTTGGCTCCACCCT
