# Supplementary material for: OCT4 impedes cell fate redirection by the melanocyte lineage master regulator MITF in mouse ESCs
Source: Nat Commun. 2017 Oct 18;8:1022. doi: 10.1038/s41467-017-01122-1 (PMC5647326; doi:10.1038/s41467-017-01122-1)
Supplement: Supplementary file 3 — Description of Additional Supplementary Files [file 41467_2017_1122_MOESM3_ESM.pdf]

Description of Additional Supplementary Files

File Name: Supplementary Data 1
Description: Differential gene expression, Related to Figure 2 d.

File Name: Supplementary Data 2
Description: Gene expression_BioGPS, Related to Figure 3 d.

File Name: Supplementary Data 3
Description: Oct4_lineage TFs_overlapping genes, Related to Figure 5 b.

File Name: Supplementary Data 4
Description: Oct4_lineage TFs_nonoverlapping genes, Related to Figure 5 b.

File Name: Supplementary Data 5
Description: MITF, E2F7 and P53 overlapped genes, Related to Supplementary Figure 4 a-d.

File Name: Supplementary Data 6
Description: OCT4_MITF_Shared domains, Related to Figure 5 f.

File Name: Supplementary Data 7
Description: Enhancers_MITF with OCT4, P53, Related to Figure 4 g.

File Name: Supplementary Data 8
Description: GO_SOX2_MITF_HES1_GATA4_CDX2, Related to Discussion.

File Name: Supplementary Data 9
Description: GO_NANOG_MITF_HES1_GATA4_CDX2, Related to Discussion.
